# Gut microbial involvement in Alzheimer's disease pathogenesis

**DOI:** 10.18632/aging.202994

**Published:** 2021-05-10

**Authors:** Yu Zhang, Rulin Geng, Qiuyun Tu

**Affiliations:** 1 Department of Geriatrics, The Fifth Affiliated Hospital of Sun Yat-Sen University, Zhuhai 519000, China

**Keywords:** Alzheimer’s disease, gut microbiota, microbiota-gut-brain axis, pathogenesis

## Abstract

Alzheimer's disease (AD) is a chronic, progressive neurodegenerative disease characterized by memory loss, inability to carry out everyday daily life, and noticeable behavioral changes. The essential neuropathologic criteria for an AD diagnosis are extracellular β-amyloid deposition and intracellular accumulation of hyperphosphorylated tau. However, the exact pathogenic mechanisms underlying AD remain elusive, and current treatment options show only limited success. New research indicates that the gut microbiota contributes to AD development and progression by accelerating neuroinflammation, promoting senile plaque formation, and modifying neurotransmitter production. This review highlights laboratory and clinical evidence for the pathogenic role of gut dysbiosis on AD and provides potential cues for improved AD diagnostic criteria and therapeutic interventions based on the gut microbiota.

## INTRODUCTION

AD is a common degenerative disease of the central nervous system (CNS). It is the most common cause of dementia and one of the greatest health-care challenges of the 21st century [1]. Global dementia costs were estimated at US$ 957.56 billion in 2015 and are projected to reach US$ 2.54 trillion in 2030 and US$ 9.12 trillion in 2050, likely exceeding the predictions of the World Alzheimer Report 2015 [2]. AD’s primary clinical manifestation is a progressive decline in memory and thinking skills, which severely interferes with daily life. Studies suggest that AD pathogenesis is diverse, and three main hypotheses (i.e. amyloid, tau protein, and neuroinflammation) have been proposed in such regard [3]. The amyloid hypothesis is the mainstream theory of AD pathogenesis and is supported by AD’s key pathological feature, namely the presence of senile plaques defined by extracellular deposition of neuritic amyloid β-protein (Aβ) [4]. Aβ peptides are produced by cleavage of amyloid precursor protein (APP) by β- and γ-secretases; Aβ production is in turn prevented by α-secretase activity, which cleaves APP within the Aβ domain [5]. In AD patients' brains, Aβ peptides are typically arranged in β sheet conformations in the form of higher-order oligomers, protofibrils, and fibrils [6]. The tau protein hypothesis is backed by increasing evidence that suggests that tau hyperphosphorylation may be a significant driving factor of neurodegeneration in AD. Tau is a microtubule-associated protein (MAP) that promotes tubulin aggregation into microtubules, stabilizing the neuronal cytoskeleton to assure proper axonal transport, neurite outgrowth, and synaptic plasticity. Tau hyperphosphorylation contributes to the disassembly of microtubules, which leads to the impairment of neuronal and synaptic structures and the formation of nerve fiber tangles [3]. The neuroinflammation hypothesis of AD focuses on the innate immune response triggered by binding of misfolded and aggregated proteins to pattern recognition receptors on microglia and astroglia, which determines the release of inflammatory mediators contributing to disease progression and severity [7]. Notably, new research suggests that in addition to amyloid deposition, tau hyperphosphorylation, and neuroinflammatory mechanisms, imbalances in the intestinal flora (gut dysbiosis) may also increase the risk of AD and contribute to its development.

The gut microbiota encompasses the microorganisms, mostly bacteria, living in the intestinal tract of animals. There are about 100 trillion bacteria (approximately ten times the number of human cells), represented by 1,000~1,500 species, in the human alimentary canal [8]. Bacteroidetes and Firmicutes account for more than 90% of all gastrointestinal bacteria in healthy humans [9]. The gut microbiota plays a vital role in synthesizing amino acids and vitamins, metabolizing steroid molecules and bioactive compounds, and strengthening the immune system [10]. Although human gut microbes are typically homeostatic, the composition of the intestinal flora changes with age. Indeed, several studies have found that age-related processes can influence gut microbiota diversity and lead to metabolic alterations with potentially deleterious effects [11–14]. Research has shown that the diversity of the intestinal flora in the elderly is decreased, with a general trend towards decreased Bifidobacterium and increased Enterococcus species abundances. Also, both age-related decline in immune function (immune senescence) and a parallel increase in intestinal permeability further affect the composition and distribution of the intestinal flora [15]. Bifidobacterium and Lactobacillus are the two most essential probiotics in the human intestine, serving to maintain a healthy intestinal environment and to regulate the immune function. Remarkably, differences in gut microbiota composition importantly determine an individual’s biochemical processes, influence epigenetic changes, and modulate psychological and cognitive functioning as well as susceptibility and resistance to disease [16, 17]. Mounting evidence links intestinal dysbiosis, resulting from external (environment) or internal (host-related) factors, with onset and progression of local inflammatory reactions and even systemic diseases such as AD, hypertension, diabetes, and depression [18–20]. In recent years, the relationship between the gut microbiota and AD has been addressed in clinical studies as well as in several animal models. Zhuang et al. compared the intestinal flora of 43 AD patients with that of 43 age- and sex-matched controls. Compared to the control group, a subgroup of inflammation-associated bacteria, including members of the Escherichia and Shigella taxa, was found to be increased in the AD cohort [21]. Vogt et al. have observed a significant decrease in the species diversity of intestinal flora from AD patients, with decreased abundance of Firmicutes and Bacilli and overrepresentation of the Bacteroides genus. In addition, a positive correlation was detected between cerebrospinal fluid (CSF) levels of YKL-40, an inflammatory biomarker associated with neurodegeneration, and the number of Bacteroides and Clostridium in the gut [22]. Shen et al. studied the relationship between gut microbiota and age in APP/PS1 transgenic and wild-type mice aged three, six, and eight months. They found that age-related changes in gut microbiota composition were associated with amyloid plaque burden and both spatial learning and memory impairment [23]. The results of the above studies suggest a correlation between gut microbiota and AD.

The microbiota-gut-brain axis entails a two-way communication system involving cytokine, immune, hormonal, and neuronal signals that is currently the focus of intensive research [24, 25]. Experimental models have been essential for unmasking gut microbiota’s regulatory actions on brain functions such as learning and memory [26]. Moreover, some studies have found that the gut microbiota can influence AD's typical pathological features, such as deposition of Aβ, hyperphosphorylation of tau, and neuroinflammation through the microbiota-gut-brain axis [27]. In this review, we examine the roles of the gut microbiota in both aging and AD and provide an update on the mechanisms by which the gut microbiota influences the pathobiology of AD. In addition, we discuss some new therapeutic interventions based on gut microbiota manipulation that might provide clinical benefit for patients |with AD.

## The pathogenic role of gut microbiota in AD

**Figure 1:**
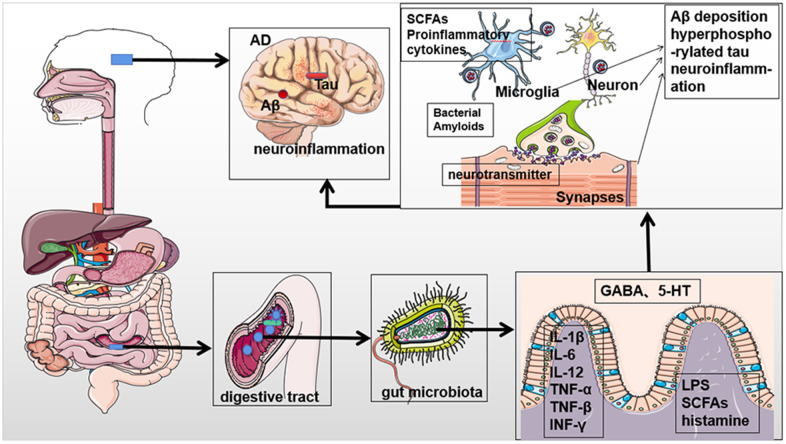
**Potential contribution of the gut microbiota to the pathogenesis of Alzheimer's disease.** The gut microbiota can affect the occurrence and progression of AD through metabolites, neurotransmitters, and proinflammatory mediators to promote Aβ aggregation, accumulation of hyperphosphorylated tau, and chronic neuroinflammation. Parts of the figure are adapted from SMART (Servier Medical Art: https://smart.servier.com), licensed under a Creative Common Attribution 3.0 Generic License.

As mentioned above, several studies support the association between gut microbiota and AD. Collectively, available data indicates that gut microbiota composition and activity can promote the occurrence of AD through many pathways, including metabolites, neurotransmitters, chronic neuroinflammation, etc. (Figure 1 and Table 1).

**Table 1:** Pathogenic relationships between gut microbiota and AD.

**Factor(s)**	**Study subjects**	**Main effects**	**References**
Bacterial abundance and anti-inflammatory factor levels	AD patients	Increased Escherichia/Shigella leading to increased proinflammatory factors	Cattaneo et al. [30]
Dysbacteriosis and neuroinflammation	5×FAD AD mouse model	Increased phenylalanine isoleucine stimulated Th1 cell differentiation and proliferation; activated microglia	Wang et al. [32]
Immune cells and neuroinflammation	AD Drosophila model	Stimulated immune hemocyte recruitment to the brain; exacerbated AD progression	Wu et al. [33]
LPS	AD patients	Increased brain contents of E. coli K99 protein and LPS; LPS can be used as a marker of AD	Zhao et al.; Zhan et al. [37–39]
Histamine	Rat primary microglia	Increased NO levels and stimulated neuroinflammation	Dong et al. [43]
SCFAs	Germ-free AD mice model	Regulated energy balance; promoted colon cell metabolism and had a powerful anti-inflammatory effect	Alessio et al. [47]
LPS and neuroinflammation	Mouse model of episodic systemic inflammation	Reduced exploratory activity and episodic and spatial memories	D' Avila et al. [55]
Peripheral inflammation	UC rat model	Elevated inflammatory markers and increased intracellular inducible NO synthase and intercellular adhesion molecules; microglial activation and astroglial loss	Villaran et al. [56]
GABA	Autopsy study of AD patients	Correlation between GABA deficiency and AD occurrence	Solas et al. [58]
Lactobacillus ingestion/GABA	BALB/c mice	Reduced stress-induced corticosterone and anxiety-like behavior	Bravo et al. [59]
5-HT and SSRI	AD transgenic mouse models	Decreased Aβ; activated α-secretase; inhibition of APP conversion into Aβ	Sharma et al. [61]
Gut microbiota and Aβ	APP_SWE_/PS1_ΔE9_ mice	Regulate host innate immunity mechanisms that impact Aβ amyloidosis.	Minter et al. [64]
tau	Male mice	Inflammation-related factors such as IL-1b, IL-6, IL-10, and TNF-α accelerated tau hyperphosphorylation	Savignac et al. [73]

Abbreviations: AD: Alzheimer’s disease; 5xFAD: 5xFAD (APP K670N, M671L, I716V, PS1 M146L, L286V) mice. Th1: T helper 1 cells; LPS: lipopolysaccharide; NO: nitric oxide; SCFAs: short-chain fatty acids; GABA: gamma-aminobutyric acid; GF: germ-free; UC: ulcerative colitis; TLR2: Toll-like receptor 2; 5-HT:5-hydroxytryptamine; SSRI: selective serotonin reuptake inhibitor.

## Gut microbiota and neuroinflammation

Mounting evidence suggests that neuroinflammation plays a vital role in AD's pathogenetic mechanisms by aggravating AD features, including Aβ deposition and tau hyperphosphorylation, further triggering inflammatory responses and leading to a vicious circle of tissue destruction and inflammation [27, 28]. Significantly increased concentrations of pro-inflammatory cytokines, including interleukin (IL)-1β, IL-6, IL-12, IL-18, tumor necrosis factor (TNF)-α, TNF-β, and interferon (INF)-γ, have been detected in the brains of AD patients [27]. Interestingly, however, depending on its nature and magnitude, inflammation can be either beneficial or harmful to the brain. Whereas chronic inflammation mediated by mild TLR4 stimulation can reduce tau hyperphosphorylation by activating neuronal autophagy, a more extensive inflammatory status can significantly promote AD [29].

Clinical research conducted by Cattaneo et al. showed that the abundance of pro-inflammatory taxa (Escherichia/Shigella) was increased, the representation of anti-inflammatory taxa (Eubacterium spp., Bacteroides fragilis) was decreased, and serum proinflammatory factors were significantly elevated in AD patients [30]. Population shifts in the gut microbiota can promote intestinal inflammation which would, over time, increase bacterial colonization and disrupt gut microbiota's dynamic balance [30, 31]. Moreover, a growing body of animal studies has also verified the relationship between gut microbiota and neuroinflammation. Wang et al. have found that alterations in gut microbiota in the 5×FAD AD mouse model lead to increased phenylalanine and isoleucine levels in peripheral blood, which stimulate the differentiation and proliferation of proinflammatory T helper 1 (Th1) cells. Following brain infiltration, Th1 cells promote microglial activation, leading to AD-related neuroinflammation and cognitive impairment [32]. Along these lines, using a Drosophila AD model, Wu et al. demonstrated that gut bacterial infection stimulates the recruitment of immune cells into the brain, exacerbating AD progression [33]. Furthermore, a study has implicated that supplementing the gut with Akkermansia muciniphila improves intestinal barrier function and increases about 3-fold the thickness of the colonic mucus layer in mice. Moreover, compared to control animals, a significant reduction in the expression of inflammation-related genes and pathways, as well as in B cell abundance, was observed in the colon of mice supplemented with A. muciniphila [34].

Altogether, this evidence confirms a close relationship between gut dysbiosis and exacerbation of the host’s inflammatory state, resulting from increased intestinal permeability and enhanced translocation of bacterial metabolites into the circulation. This triggers proinflammatory cytokine production and inflammatory responses that may disrupt blood-brain-barrier (BBB) integrity, facilitating the onset of nervous system diseases characterized by neurodegeneration and cognitive/behavioral deficits such as AD, Parkinson’s disease, frontotemporal dementia syndromes, and amyotrophic lateral sclerosis, among others.

## Impact of gut microbiota metabolites on AD

Under physiological conditions, the gut microbiota produces lipopolysaccharide (LPS), short-chain fatty acids (SCFAs), histamine, and other metabolites that can potentially affect the central nervous system (CNS). The intestinal mucosal barrier blocks the transfer of harmful substances into the circulation, protecting mucosal tissue and the circulatory system from microbes, microbial toxins, and other pro-inflammatory substances. However, when the tight junctions that maintain intestinal mucosal barrier function are disrupted, intestinal leakage ensues and can induce various diseases. The permeability of both the intestinal mucosal barrier and the BBB also increase during physiological aging. Through high-performance liquid chromatography analysis, Leblhuber et al. have found that the concentration of fecal calreticulin in the blood of AD patients is significantly increased, indicating that calreticulin can cross the intestinal barrier and reach the circulatory system to induce neuroinflammation [35, 36]. It is now well acknowledged that intestinal metabolites can cross the BBB and damage neuronal function, contributing to AD pathogenesis. However, not all intestinal metabolites are harmful to the nervous system. For instance, some metabolites, such as SCFAs (see below), can improve microglial function, aiding in the removal of brain debris, including neuritic plaques and other aggregates.

The gut microbiota is an important source of LPS, a potent proinflammatory mediator produced by Gram-negative bacteria. Zhao et al. were the first to report the presence of LPS in the hippocampus and neocortex of the superior temporal lobe of AD patients. Compared with the control group, hippocampal LPS levels in elderly AD patients exhibited up to a 26-fold increase [37]. In turn, applying immunoblotting and immunohistochemistry, Zhan et al. further showed that E. coli K99 protein and LPS levels were higher in the brains of AD patients, compared to non-AD controls [38, 39]. Histamine is produced by enterochromaffin-like cells in the stomach, by mast cells and basophils as part of an immune response, and is also a product of intestinal microbial metabolism. Lactobacillus, Lactococcus, and Streptococcus, among other bacteria, possess histidine decarboxylase genes and can produce histamine [40]. Histamine is a physiological regulator of cell proliferation, allergic reactions, and immune cell function and acts as a neurotransmitter in the brain [41]. High-density histamine receptors on neurons in the striatum, thalamus, amygdala, and other regions indicate the extensive role of histamine in the CNS. It has been found that increases in histamine levels are related to AD and may increase nitric oxide production and stimulate neuroinflammation. This is consistent with the hypothesis that low-grade inflammation contributes to the development of neurodegenerative diseases [42, 43]. SCFAs are produced mainly by gut microbiota processing of undigested fibres and proteins. SCFAs have a beneficial effect on the host, especially in regulating systemic metabolism and energy balance. In addition, SCFAs play a key role in promoting colon cell metabolism and possess a powerful anti-inflammatory effect [44, 45]. Previous research suggested that SCFAs can inhibit the aggregation of Aβ *in vitro* [46]. Brain microglia are essential for clearance of protein aggregates, such as senile plaques, and several studies have found that SCFAs produced by microbes can improve damaged microglia function in germ-free (GF) animals [47]. Yuan et al. used isotope labelling reagents to label metabolites of different chemical groups and applied liquid chromatography-mass spectrometry to establish a database of fecal metabolomics in mice. Through similar analyses, the identification of metabolic changes in AD patients' stool may yield new biomarkers for AD diagnosis [48, 49].

## Intestinal metabolites and neuroinflammation

Mounting evidence links gut dysbiosis with neuroinflammation, an essential feature of the pathogenesis of AD. With increasing age, the permeability of the intestinal epithelium increases, promoting the translocation of Gram-negative bacteria and LPS into the blood [50]. Locally, Gram-negative bacteria invade the intestinal lamina propria and mesenteric lymph nodes and stimulate immune cells to release proinflammatory factors into the systemic circulation, leading to an intestinal inflammatory response that further increases intestinal and BBB permeability. This facilitates the entry of LPS into the brain, which triggers neuroinflammation by activating Toll-like receptors (TLRs) in microglia, thus favoring the development of AD [51, 52]. Zhao et al. showed that LPS contents in the neocortex and the hippocampus of AD patients showed a prominent perinuclear localization and were 2- and 3-fold higher, respectively, than those measured in samples from age-matched, healthy controls [39, 54]. In turn, it was reported that addition of LPS from Bacteroides fragilis (a major Gram-negative bacillus of the human gastrointestinal tract [53]) to a co-culture system of human neuronal and glial cells led to significant suppression of several synapsis-associated proteins, including NRXN1, SNAP25, SYN2, NLGN, and SHANK3 [54]. d' Avila et al. administered a low-dose LPS regimen to young (2 months old) and aged (12 months old) mice and noted that the aged mice were more susceptible to sporadic systemic inflammation and showed also reduced exploratory activity and significantly decreased episodic and spatial memory functions [55]. Further evidence that peripheral inflammation induced by intestinal metabolites promotes the occurrence and development of neuroinflammation was provided by Villarán et al., who found that dopaminergic neurodegeneration induced by injection of LPS into the substantia nigra was potentiated in a rat model of ulcerative colitis. The observed changes included an increased inflammatory response defined by elevation of serum levels of inflammatory markers (TNFα, IL-1β, IL-6, and C reactive protein, increased expression of inducible NO synthase and intercellular adhesion molecules, enhanced microglial reactivity, and astrocyte death [38, 56].

## Gut microbiota-derived neurotransmitters

Stress and mood can cause the brain to influence gut microbial composition by releasing hormones and neurotransmitters. In turn, the gut flora can influence brain function by producing neurotransmitter precursors and regulating host neurotransmitter catabolism. The gut microbiota synthesizes numerous neurotransmitters, including γ-aminobutyric acid (GABA), norepinephrine, acetylcholine, and dopamine, and stimulate the secretion of 5-hydroxytryptamine (5-HT) by enterochromaffin cells in the intestinal wall [57]. GABA and 5-HT are major neurotransmitters with important roles in mood and cognition. Suggesting a correlation between GABA deficiency and AD development, Solas et al. found decreased GABA levels in the frontal, temporal, and parietal cortices of AD patients post-mortem [58]. Meanwhile, Bravo et al. found that feeding Lactobacillus rhamnosus (JB-1) to mice reduced the expression of GABA_Aα2_ mRNA in the prefrontal cortex and amygdala and decreased stress-induced corticosterone production and anxiety-like behavior. Of note, these effects were abrogated in vagotomized mice [59]. More than 95% of 5-HT is synthesized in the intestine, and intestinal bacteria can inhibit the synthesis of 5-HT. Linstow et al. analyzed 5-HT levels in neocortical, hippocampal, striatal, brainstem, and cerebellar samples from AD transgenic mice aged 18 months. The results showed region-specific changes in all monoamines in AD mice compared to wild-type mice, with 5-HT levels exhibiting a 30% reduction in the neocortex and an 18% increase in the brainstem [60]. Studies have shown that using selective serotonin reuptake inhibitors (SSRIs) or directly increasing extracellular 5-HT significantly reduced (by 25%) Aβ content in brain interstitial fluid in mice. One of the mechanisms by which SSRIs decrease Aβ formation is by activating α secretase activity, which inhibits APP conversion into Aβ. Upon binding to 5-HT receptors, SSRI can also reduce Aβ aggregation by inhibiting 5-HT reuptake, thus increasing 5-HT concentration in the brain. Along these lines, it was reported that chronic (over four months) SSRI administration decreased Aβ plaque load by 50% in AD transgenic mice [61].

Given the tight relationship between intestinal flora composition, 5-HT production, and Aβ aggregation, new treatments that focus on restoring gut microbiota balance might, in combination with SSRIs, prove to be effective in normalizing brain 5-HT concentrations and attenuating Aβ aggregation in AD.

## Impact of the gut microbiota on cerebral amyloid and tau proteins

The gut microbiota can affect the deposition of Aβ in the brain in a variety of ways. Proinflammatory cytokines can enhance the expression of APP and promote the formation of Aβ in the hippocampus. Multiple bacterial genera, such as Streptomyces, Staphylococcus, Bacillus, and Escherichia secrete functional amyloid proteins, similar in both structure and immunogenicity to the human Aβ42 peptide, which may act as pathogen-associated molecular pattern recognition molecules. Bacterial amyloids may promote AD by binding to microglial TLR2, leading to microglial activation through upregulation of Notch1 signaling [62–66]. A finding suggests the gut microbiota community diversity can regulate host innate immunity mechanisms that impact Aβ amyloidosis [67]. In turn, Pistollato et al. reported that soluble Escherichia coli LPS monomer can accelerate the polymerization of Aβ monomers into insoluble aggregates [68]. In summary, bacteria-derived products such as LPS and amyloid can promote AD through both direct (enhanced amyloid deposition) and indirect (TLR-mediated inflammation) mechanisms.

Neurofibrillary tangles formed by hyperphosphorylated tau are another major pathological characteristic of AD. Tau phosphorylation decreases its affinity for microtubules and causes microtubule disassembly; this compromises the structural integrity of neurons, leading eventually to their death [69]. Gut microbes can also increase or decrease tau phosphorylation in a variety of ways. The best-defined ones are related to oxidative stress, inflammation, and regulation of autophagy. Some beneficial effects of gut microbes on tau phosphorylation dynamics are exemplified by Clostridium sporogeneses, which uses tryptophan to produce the strong antioxidant 3-indole propionic acid, and by lactic acid bacteria, which synthesize metabolic factors that significantly improve the activity of SOD and GSH-Px, thus reducing oxidative stress and tau aggregation [70–72]. Savignac et al. designed a rat model of peripheral infection by tail vein injection of LPS. Results showed that inflammation-related factors such as IL-1b, IL-6, IL-10, and TNF-α accelerated tau hyperphosphorylation [73].

## Conclusions and future perspectives

AD, a neurodegenerative disease, is the most prevalent cause of dementia and a serious threat to human health and life quality. As AD incidence continues to increase, intensified research heralds renewed hope for more effective treatments. Although recent studies unequivocally affirm a close association between gut microbiota imbalances and cognitive impairment in AD patients, there is still a knowledge gap about the mechanisms behind these linkages. Nevertheless, the data available have important implications for future AD research directions, opening the door to novel AD diagnostic criteria and therapeutic interventions that consider, respectively, relevant alterations and targeted modulation of the gut microbiota. Still, given the considerable overlap between gut microbiota profiles from elderly individuals with and without AD (Table 2), further research on the mechanisms underlying the link between AD and gut microbiota is warranted to clearly identify candidate microbial biomarkers for AD diagnosis and treatment. In this regard, comparative studies addressing not only composition, but also metabolic activity of gut bacteria in AD and non-AD individuals should be conducted to determine the best microbial candidates to guide clinical treatment.

**Table 2:** Gut microbiota alterations in elderly controls and AD patients.

**Bacterial species**	**Alterations of abundance**		**Mechanisms**	**References**
	**Elderly**	**AD patients**		
Clostridiales IV	↓	↓	Butyric acid is associated with resistance to inflammation and aging	Liu et al. [74]
Clostridiales X, α	↓	↓↓	Norepinephrine, acetylcholine and other neurotransmitters are related to cognitive and memory functions	Wall et al. [75]
Lactobacillus	↓	↓↓	Conversion of glutamate to GABA. Cognitive impairment may be due to disorders of the GABA system	Zhuang et al. [21]
Bifidobacterium	↓	↓↓	Bacteria can provide energy through SCFA, which can be used to promote the synthesis and secretion of neurotransmitters and hormones and to reduce the inflammatory response	Vogt et al. [22]
Staphylococcus aureus	↑	↑↑	Bacteria can secrete Aβ. Abnormal accumulation of Aβ activates diverse cellular receptors, leading to release of inflammatory factors which trigger or intensify the inflammatory response	Zhao et al. [62]
Escherichia coli	↑	↑↑	Bacterial metabolites exacerbate peripheral inflammation and can promote Aβ aggregation and cytotoxicity	Radli et al. [76]
Cyanobacteria	↑	↑↑	The neurotoxic amino acid BMAA causes protein misfolding and is a possible mechanism for β-amyloid deposition in AD patients	Banack et al. [77]
Gram-negative bacteria	↑	↑↑	LPS production stimulates the release of many inflammatory factors, promoting an inflammatory response	Itzhaki et al. [78]
Streptococcus	/	↓	Promotion of disease through immune mechanisms	Li et al. [79]
Bacteroides fragilis, Eubacterium spp.	/	↓	Reduced anti-inflammatory protection	Cattaneo et al. [30]

Abbreviations: AD: Alzheimer disease; GABA: gamma-aminobutyric acid; SCFA: short-chain fatty acid; Aβ: β amyloid; BMAA: β-methylamino-L-alanine.

Whereas the brain controls intestinal movement and secretion by regulating autonomic nerve function, the gut microbiota can also affect neurogenic control of intestinal functions by influencing neurogenesis and neuronal activity. The evidence collected on the microbiota-gut-brain axis suggests that gut microbes can also regulate AD onset and progression by affecting the integrity of the BBB, nerve growth, neurotransmitter production, and microglial activity. During gut dysbiosis, noxious signals are transmitted from the gut to the brain, resulting in chronic low-grade inflammation, oxidative stress, and cellular dysfunction and degeneration. Cytokines, neurotransmitters, and metabolites produced by gut microbiota or induced by bacterial factors in host cells can cross the BBB and impair neuronal function. However, not all microbial metabolites negatively affect the nervous system. For instance, SCFAs protect the intestinal barrier by preserving the integrity of the intestinal endothelium, which reduces the risk of peripheral inflammation [80, 81]. Moreover, research has shown that SCFAs can effectively inhibit Aβ aggregation *in vitro*, which supports a possible protective effect of the intestinal flora on AD. Still, to materialize the potential of gut microbiota’s compositional and metabolic profiling as an aid to AD diagnosis, large-scale multi-center studies are needed to address the potentially large variability arising from patients' dissimilar geographical location, diet, living habits, and comorbidities (.e.g. gastrointestinal disorders). For example, compared to healthy controls, a lower abundance of Bacteroides was found in fecal samples of AD patients in a Chinese cohort, but an increase in Bacteroides abundance was reported in AD patients from the USA [74, 82]. The current diagnosis of AD requires CSF biomarker analysis and brain imaging in addition to cognitive scale assessment [83, 84]. The most advantageous feature of gut microbiota analysis in AD patients is the possibility of providing a non-invasive diagnostic approach that should increase patient compliance. At present, the urgent problem is distinguishing which aspects of the complex pathogenicity of AD are directly regulated by intestinal microbes through mutual interactions with the host, and which constitute in turn host's inherent factors. Moreover, our knowledge about gut microbiota effects on AD pathogenesis is still limited to various findings regarding cellular events, whereas much less is known about the molecular and genetic underpinnings. Hence, further research is needed to develop uniform criteria for clinical testing.

Given the close relationship evidenced between gut microbes and AD, and the fact that there is still no effective treatment for AD [85], the regulation of gut microbiota has attracted wide attention as potential therapeutic strategy [86]. Current AD treatments include probiotics, fecal transplantation, antibiotics, and dietary adjustments [87–89]. Probiotics can reduce Aβ deposition by regulating gut microbiota homeostasis, reducing the excessive release of anti-inflammatory factors, and improving cognitive function. However, further experiments are indispensable to clarify the specific clinical strains and dosage cycles to be applied. It has been reported that fecal bacteria transplantation can reduce amyloid deposition and improve memory in the AD mouse model, but a potential deficiency of this technique is that it may increase the risk of infection with pathogenic microorganisms. Some clinical and animal experiments showed that antibiotics could alleviate AD symptoms and disease processes by influencing neurotransmitter dynamics, oxidative stress mechanisms, and by diminishing neuroinflammatory processes [90, 91]. However, most of the existing studies used broad-spectrum antibiotics, which are associated with the development of drug resistance. The negative effects of antibiotics can be alleviated through concomitant treatment with selected probiotics. Nevertheless, antibiotics with selective antibacterial effects would still be preferable, which stresses the need to identify in AD patients specific changes in gut microbiota composition and metabolic activity. In turn, the probiotics' true status as AD therapeutic agents depends on more conclusive research on gut microbiota's role as a disease regulator. Therefore, before clinical implementation of the above treatment strategy, pre-clinical trials are needed to verify its feasibility and efficacy.

In summary, a growing body of evidence reveals the link between gut microbiota and AD. However, since the underlying pathological mechanisms require further scrutiny, therapeutic application of gut microbiota-targeted approaches remains so far at an experimental stage. Predictably, however, intestinal microecological intervention studies will provide a renewed understanding of the gut microbiota’s role on AD and allow the development of more effective strategies to prevent and treat this condition.

## Additional information

Correspondence and requests for materials should be addressed to Q.Tu.
